# Glucose regulates tissue-specific chondro-osteogenic differentiation of human cartilage endplate stem cells via *O*-GlcNAcylation of Sox9 and Runx2

**DOI:** 10.1186/s13287-019-1440-5

**Published:** 2019-11-28

**Authors:** Chao Sun, Weiren Lan, Bin Li, Rui Zuo, Hui Xing, Minghan Liu, Jie Li, Yuan Yao, Junlong Wu, Yu Tang, Huan Liu, Yue Zhou

**Affiliations:** 1 Department of Orthopedics, Xinqiao Hospital, Army Medical University, Chongqing, 400038 People’s Republic of China; 2 Department of Orthopaedics, The Second Affiliated Hospital of Southwest Medical University, Lu Zhou, 646000 Sichuan People’s Republic of China

**Keywords:** Glucose, *O*-GlcNAcylation, Cartilage endplate stem cells, Chondrogenic differentiation, Osteogenic differentiation

## Abstract

**Background:**

The degenerative disc disease (DDD) is a major cause of low back pain. The physiological low-glucose microenvironment of the cartilage endplate (CEP) is disrupted in DDD. Glucose influences protein *O*-GlcNAcylation via the hexosamine biosynthetic pathway (HBP), which is the key to stem cell fate. Thiamet-G is an inhibitor of *O*-GlcNAcase for accumulating *O*-GlcNAcylated proteins while 6-diazo-5-oxo-l-norleucine (DON) inhibits HBP. Mechanisms of DDD are incompletely understood but include CEP degeneration and calcification. We aimed to identify the molecular mechanisms of glucose in CEP calcification in DDD.

**Methods:**

We assessed normal and degenerated CEP tissues from patients, and the effects of chondrogenesis and osteogenesis of the CEP were determined by western blot and immunohistochemical staining. Cartilage endplate stem cells (CESCs) were induced with low-, normal-, and high-glucose medium for 21 days, and chondrogenic and osteogenic differentiations were measured by Q-PCR, western blot, and immunohistochemical staining. CESCs were induced with low-glucose and high-glucose medium with or without Thiamet-G or DON for 21 days, and chondrogenic and osteogenic differentiations were measured by Q-PCR, western blot, and immunohistochemical staining. Sox9 and Runx2 *O*-GlcNAcylation were measured by immunofluorescence. The effects of *O*-GlcNAcylation on the downstream genes of Sox9 and Runx2 were determined by Q-PCR and western blot.

**Results:**

Degenerated CEPs from DDD patients lost chondrogenesis, acquired osteogenesis, and had higher protein *O*-GlcNAcylation level compared to normal CEPs from LVF patients. CESC chondrogenic differentiation gradually decreased while osteogenic differentiation gradually increased from low- to high-glucose differentiation medium. Furthermore, Thiamet-G promoted CESC osteogenic differentiation and inhibited chondrogenic differentiation in low-glucose differentiation medium; however, DON acted opposite role in high-glucose differentiation medium. Interestingly, we found that Sox9 and Runx2 were *O*-GlcNAcylated in differentiated CESCs. Finally, *O*-GlcNAcylation of Sox9 and Runx2 decreased chondrogenesis and increased osteogenesis in CESCs.

**Conclusions:**

Our findings demonstrate the effect of glucose concentration on regulating the chondrogenic and osteogenic differentiation potential of CESCs and provide insight into the mechanism of how glucose concentration regulates Sox9 and Runx2 *O*-GlcNAcylation to affect the differentiation of CESCs, which may represent a target for CEP degeneration therapy.

## Background

Degenerative disc disease (DDD) plays an important role in back pain, which can cause activity limitation [1, 2]. Inflammation, cell senescence, cell death, mechanical injury, and other pathological factors can lead to DDD [3–5]. The main mechanism of DDD is decreased nutrition of the disc, leading to cell waste product accumulation, microenvironment disturbance, and matrix molecule degradation that further disrupts cell activities [6]. The intervertebral disc (IVD) is avascular, and it receives nutrition from the surrounding vasculature. Poor nutritional supply of the IVD is an important factor in the pathophysiology of DDD. The cartilage endplate (CEP) plays a critical role in the metabolic exchange in the IVD [7, 8]. The CEP is a thin horizontal layer of hyaline cartilage, and it separates the IVD from the vertebral body. Blood vessels from the adjacent bones just reach the interface between the IVD and vertebrae [9]. CEP degeneration and insufficient nutrition supply may initiate DDD [10].

The CEP belongs to hyaline cartilage, the matrix of it is composed of collagen type II (COL2), and its physiological function is chondrogenesis characteristics. But CEP calcification is one major pathological character in IVD degeneration [11]. CEP calcification induced degeneration by inhibiting obstructing nutrient and oxygen supply in IVD [12]. So, CEP chondrogenesis converts into osteogenesis during the IVD degeneration. However, the mechanisms are unclear.

Our group isolated and identified stem cells in human CEP firstly. The cartilage endplate stem cells (CESCs) were similar to bone marrow mesenchymal stem cells (BM-MSCs), and they had a stronger potential of chondrogenic and osteogenic differentiation than BM-MSCs (Additional file 1: Figure S1) [13]. The normal CEP in healthy human is composed of cartilage, while the composition transformed to bone in degeneration CEP. Therefore, CESCs may play a critical role in the chondrogenesis and osteogenesis of CEP.

As an avascular tissue, IVD remains in a low-glucose microenvironment [14], with the concentration of glucose inside CEP is as low as 1 mM, and outside of CEP is nearly that of blood glucose approximately 5 mM [15]. Therefore, glucose greatly affects the chondrogenesis and osteogenesis of CESCs, which indicates that physiological glucose may regulate the chondro-osteogenic differentiation of CESCs to maintain a balance of chondrogenesis and osteogenesis in CEP.

The hexosamine biosynthetic pathway (HBP), an important signaling pathway in response to glucose, could mediate the connection between glucose flux, cellular signaling, and cell differentiation [16, 17]. The intracellular protein *O*-GlcNAcylation is regulated by extracellular glucose through HBP. The glutamine-fructose-6-phosphate transaminase (GFPT), a rate-limiting enzyme of HBP, could be inhibited by 6-diazo-5-oxo-l-norleucine (DON) [18, 19]. *O*-linked β-*N*-acetylglucosamine (O-GlcNAc) is composed of an *O*-β-glycosidic and a single *N*-acetylglucosamine (GlcNAc), and it acts on the serine and threonine residues of nuclear or cytoplasmic proteins [20]. *O*-GlcNAc transferase (OGT) and *O*-GlcNAcase (OGA) regulate *O*-GlcNAcylation: a single *O*-GlcNAc residue is added to proteins with the catalysis of OGT [21], and OGA removes the *O*-GlcNAc from nuclear or cytoplasmic proteins [22]. Thiamet-G could acutely augment *O*-GlcNAc levels by inhibiting OGA [23]. A lot of cellular activities, such as cell signaling, transcription, translation, stress response, and stem cell differentiation, were regulated by changing in *O*-GlcNAc modification [24, 25]. And *O*-GlcNAcylation also has involvement in a range of diseases, including Alzheimer’s disease, cancers, and diabetes [26, 27]. However, the impact of the HBP and *O*-GlcNAcylation on chondro-osteogenic differentiation is rarely reported. The transcription factor Sox9 is the master regulator of chondrocyte differentiation and skeletal development [28]. The transcription factor Runt-related transcription factor 2 (Runx2) is a member of Runt family. Runx2 is essential for osteoblast differentiation and bone development and is a key transcription factor associated with matrix formation, remodeling, and mineralization [29].

In this study, we investigated how glucose regulated Sox9 and Runx2 via *O*-GlcNAcylation, which resulted in a change in the chondro-osteogenic differentiation fate of CESCs.

## Methods

### Patients and tissue procurement

We obtained CEP tissues from patients who underwent discectomy and fusion operations at Xinqiao Hospital of Army Medical University (Table 1). We obtained normal human CEP tissues from five patients with lumbar vertebral fracture (LVF) without low back pain. The degenerated CEPs were obtained from eight patients with DDD. We used the Pfirrmann classification system to evaluate the state of IVD degeneration [30]. The surgically removed CEPs were cleaned and washed with 0.1 M sterile PBS. Then, the CEP tissues were saved for HE staining, immunohistochemistry, western blot, and CESC isolation.

**Table 1:** Patient information enrolled in this study

Case no.	Gender	Age (year)	Diagnosis	Pfirrmann grade
1	Female	53	Lumbar vertebral fracture	1
2	Male	41	Lumbar vertebral fracture	1
3	Female	39	Lumbar vertebral fracture	1
4	Female	22	Lumbar vertebral fracture	1
5	Male	31	Lumbar vertebral fracture	1
6	Female	57	Lumbar disc degeneration	6
7	Male	49	Lumbar disc degeneration	6
8	Male	44	Lumbar disc degeneration	6
9	Male	53	Lumbar disc degeneration	6
10	Female	61	Lumbar disc degeneration	6
11	Male	59	Lumbar disc degeneration	6
12	Male	63	Lumbar disc degeneration	6
13	Female	55	Lumbar disc degeneration	6

### Immunohistochemistry

The CEP tissues collected were fixed immediately with 4% formalin for 12 h and dehydrated for 48 h with 40% sucrose solution. Then, the CEP tissues were cut to 5 μm thickness and dried on glass slides for 3 h. A 3% H_2_O_2_ solution was used to stain the slides for 5 min. Then, the tissues were blocked with 3% serum and incubated with mouse anti-human *O*-GlcNAcylation antibody (RL2) (MA1-027, Thermo, MA, USA), mouse anti-human COL1 antibody (ab90395, Abcam, Cambridge, UK), and rabbit anti-human COL2 antibody (ab34712, Abcam), overnight at 4 °C. The tissue sections were washed three times in PBS and incubated in the corresponding secondary antibody (anti-rabbit immunoglobulin-horseradish peroxidase [IgG-HRP]-linked antibody, #7074, Cell Signaling Technology, MA, USA; anti-mouse IgG-HRP-linked antibody, #7076, Cell Signaling) for 1 h. Finally, nickel-diaminobenzidine images were obtained with a microscope.

### CESC isolation and culture

The CESCs were isolated from normal CEPs of five LVF patients. We cleaned and washed the surgically removed CEPs with 0.1 M sterile PBS. Then, the CEP tissues were mechanically minced and digested with 0.2% collagenase II (Sigma) in DMEM/F12 medium (Hyclone) with 1% fetal calf serum (FSC; Gibco) at 37 °C for 12 h. The suspended cells were filtered with a 70-μm cell filter and centrifuged at 200×*g* for 5 min. The pellets were resuspended in DMEM/F12, 10% FCS, and 1% penicillin-streptomycin (Hyclone) medium. Finally, cells were cultured in a 25-cm^2^ cell culture flask at 5% CO_2_ and 37 °C. The second passage cells were cultured in agarose solution [13]. The culture medium was changed twice per week. The cells were transferred in a 25-cm^2^ cell culture flask after 6 weeks. We used passage 3 cells in our study.

To induce CESC chondrogenic differentiation, the medium was then replaced with no-glucose DMEM (Thermo), 0.01% dexamethasone (Cyagen), 0.3% ascorbate (Cyagen), 1% ITS cell culture supplement (Cyagen), 0.1% sodium pyruvate (Cyagen), 0.1% proline (Cyagen), 1% TGF-β3 (Cyagen), and glucose (1 mM, 5 mM, and 25 mM) (Gibco). Then, CESCs were cultured at 37 °C and 5% CO_2_ for different periods of time up to 21 days. To induce CESC osteogenic differentiation, the medium was replaced with no-glucose DMEM (Thermo), 10% fetal bovine serum, 1% penicillin-streptomycin, 1% glutamine, 0.2% ascorbate, 1% β-glycerophosphate, 0.01% dexamethasone, and glucose (1 mM, 5 mM, and 25 mM) (Gibco). Then, CESCs were cultured at 37 °C and 5% CO_2_ for different periods of time up to 21 days.

### Induction and reduction of *O*-GlcNAcylation in CESCs during differentiation

The highly selective OGA inhibitor Thiamet-G (Cayman, MI, USA) delivered at a dose of 1 μM or PBS (vehicle) was added in low-glucose (1 mM, LG) or high-glucose (25 mM, HG) differentiation medium. To suppress the presence of UDP-GlcNAc, the GFPT inhibitor DON (Sigma) at a dose of 10^−5^ M or PBS was added in low-glucose (1 mM, LG) or high-glucose (25 mM, HG) differentiation medium. The culture medium was changed every 3 days.

### Flow cytometry

We trypsinized and washed the the CESCs. Then, the cells were stained with the following antibodies: mouse anti-human CD14-FITC (11-0149-41), mouse anti-human CD19-FITC (11-0199-41), mouse anti-human CD34-FITC (11-0349-41), mouse anti-human CD45-FITC (11-9459-41), mouse anti-human CD73-FITC (11-0739-41), mouse anti-human CD90-FITC (11-0909-41), mouse anti-human CD105-PE (12-1057-41), and mouse anti-human HLA-DR-PerCP-Cyanine 5.5 (45-9956-41), which were purchased from eBioscience (eBioscience, MA, USA). IgG antibodies (mouse IgG1 kappa isotype control-FITC, 11-4714-81; mouse IgG1 kappa isotype control-PE, 12-4714-41; mouse IgG2b kappa isotype control-PerCP-Cyanine 5.5, 45-4732-80; eBioscience, MA, USA) were used as isotype controls. The cells were incubated at 37 °C for 30 min and washed three times with PBS. Finally, CESCs were subjected to flow cytometry analysis, and the percentage of positive staining was calculated.

### Alcian blue staining and Alizarin red staining

For Alcian blue staining, we rinsed with PBS and fixed them with methanol for 3 min at − 20 °C. Then, cells were stained with 0.1% Alcian blue (Cyagen) in 0.1 M HCl for 2 h. We rinsed the stained culture plates with PBS three times and extracted them with 2 ml of 6 M HCl for 2 h at room temperature. At last, we measured the absorbance of aliquots of the extracted dye at 620 nm in a microplate reader [31].

For Alizarin red staining, cells were washed with PBS and fixed with 4% paraformaldehyde in PBS for 30 min. Staining was performed with Alizarin red (Cyagen) for 5 min at room temperature. After staining, cultures were washed three times in PBS. For quantification of Alizarin red staining, we added 10% acetic acid and incubated for 30 min with shaking vortex vigorously for 30 s, then heated to 85 °C for 10 min, transferred to ice for 5 min, and centrifuged at 20,000*g* for 15 min, and the supernatant was removed and transferred to a new 1.5-ml microcentrifuge tube, and then added with 10% ammonium hydroxide. We measured the aliquots of the supernatant in triplicate at 405 nm in 96-well format using opaque-walled, transparent-bottomed plates [32].

All images were acquired with a Leica DM4000B microscope and DFC420 camera (Leica Microsystems SAS).

### Quantitative real-time PCR

Total RNA was purified with TRIzol extraction (Invitrogen, CA, USA). Then, RNA was transcribed into cDNA using the PrimeScript RT Master Mix Kit (TaKaRa, Shiga, Japan). The primers were designed to amplify 100–250 bp sized products (see Table 2). Q-PCR was performed in triplicate in 10 μl reactions containing SYBR Premix Ex Taq II (TaKaRa). The Q-PCR samples were incubated at 95 °C for 30 s followed by 40 cycles of 95 °C for 5 s and 60 °C for 34 s and a dissociation curve analysis. The expression of each gene was normalized to GAPDH expression.

**Table 2:** Primer sequences used in this study

Primer for RT-PCR	
Gene symbol	Primer sequences (5′ to 3′)
GAPDH-F^a^	CTCTCTGCTCCTCCTGTTCG
GAPDH-R^b^	TTAAAAGCAGCCCTGGTGAC
Sox9-F	TTTGCTTGTTCACTGCAGTCTTAAG
Sox9-R	GGCATCTGCCTTCCACATG
COL2-F	GCTCCCAGAACATCACCTACCA
COL2-R	AACAGTCTTGCCCCACTTACCG
AGN-F	CACGATGCCTTTCACCACGAC
AGN-R	TGCGGGTCAACAGTGCCTATC
Runx2-F	TACAGTAGATGGACCTCGGGAAC
Runx2-R	GCGGGACACCTACTCTCATACTG
COL1-F	CACAGAGGTTTCAGTGGTTTGG
COL1-R	ACCATCATTTCCACGAGCA
ALP-F	CAAAGGCTTCTTCTTGCTGG
ALP-R	GGTCAGAGTGTCTTCCGAGG
OCN-F	CGCCTGGGTCTCTTCACTAC
OCN-R	CTCACACTCCTCGCCCTATT

*F*^*a*^ forward, *R*^*b*^ reverse

### Western blot

We harvested and lysed cells in western blot lysis buffer (Beyotime Biotechnology, Haimen, China). Then, we measured total protein concentrations with BCA kit (Beyotime Biotechnology). Equal amounts of protein were loaded and separated on a SDS-PAGE gel and transferred to a polyvinylidene fluoride membrane (Millipore Billerica, MA, USA). Then, the membranes were incubated with the corresponding primary antibodies (rabbit anti-human GAPDH, Abcam, ab181602; rabbit anti-human Sox9, Abcam, ab185230; rabbit anti-human COL2, Abcam, ab34712; rabbit anti-human AGN, Abcam, ab36861; rabbit anti-human Runx2, Cell Signaling, #12556; mouse anti-human COL1, Abcam, ab34710; rabbit anti-human ALP, Abcam, ab83259; and mouse anti-human *O*-GlcNAcyaltion antibody (RL2), Thermo, MA1-027) respectively, overnight at 4 °C, washed three times in PBS, and incubated with the corresponding secondary antibody (anti-rabbit IgG-HRP-linked antibody, #7074, Cell Signaling; anti-mouse IgG-HRP-linked antibody, #7076, Cell Signaling) for 1 h. The membranes were washed three times in PBS and subjected to western blotting using a Pierce ECL western blotting substrate kit (Thermo Scientific, MA, USA). Immunoreactive proteins were measured with chemiluminescent detection.

### Immunofluorescence

We reseeded cells in cell culture dishes. Then, we fixed cells with 4% PFA and treated them with blocking solution (1% goat serum and 0.5% Triton X-100). The cells were stained with rabbit anti-human *O*-linked *N*-acetylglucosamine (RL2) (MA1-027, Thermo) over night at 4 °C. Then, the cells were washed three times with PBS and incubated with FITC-conjugated goat anti-rabbit IgG secondary antibody (A24532, Invitrogen) for 1 h at room temperature. The cell nuclei were stained with 0.1 mg/ml DAPI (Invitrogen). Finally, the stained cells were examined with a confocal microscope.

### Immunoprecipitation

Immunoprecipitation of cell lysate components or fractionated samples was performed using Dynabeads Protein G (Invitrogen) according to the manufacturer’s instructions. We collected immunoprecipitates using magnets, suspended in SDS-sample buffer, and heated at 95 °C for 2 min. At last, the immunoprecipitates were subjected to SDS-PAGE and immunoblot analysis as described in the “Western blot” section.

### Statistical analyses

The data were expressed as the mean ± SD of independent experiments. Student’s *t* test and one-way ANOVA were used for statistical tests (*p < 0.05, **p < 0.01, and ***p < 0.001).

## Results

### Degenerated CEP in a normal glucose microenvironment lost chondrogenesis and acquired osteogenesis

**Fig. 1:**
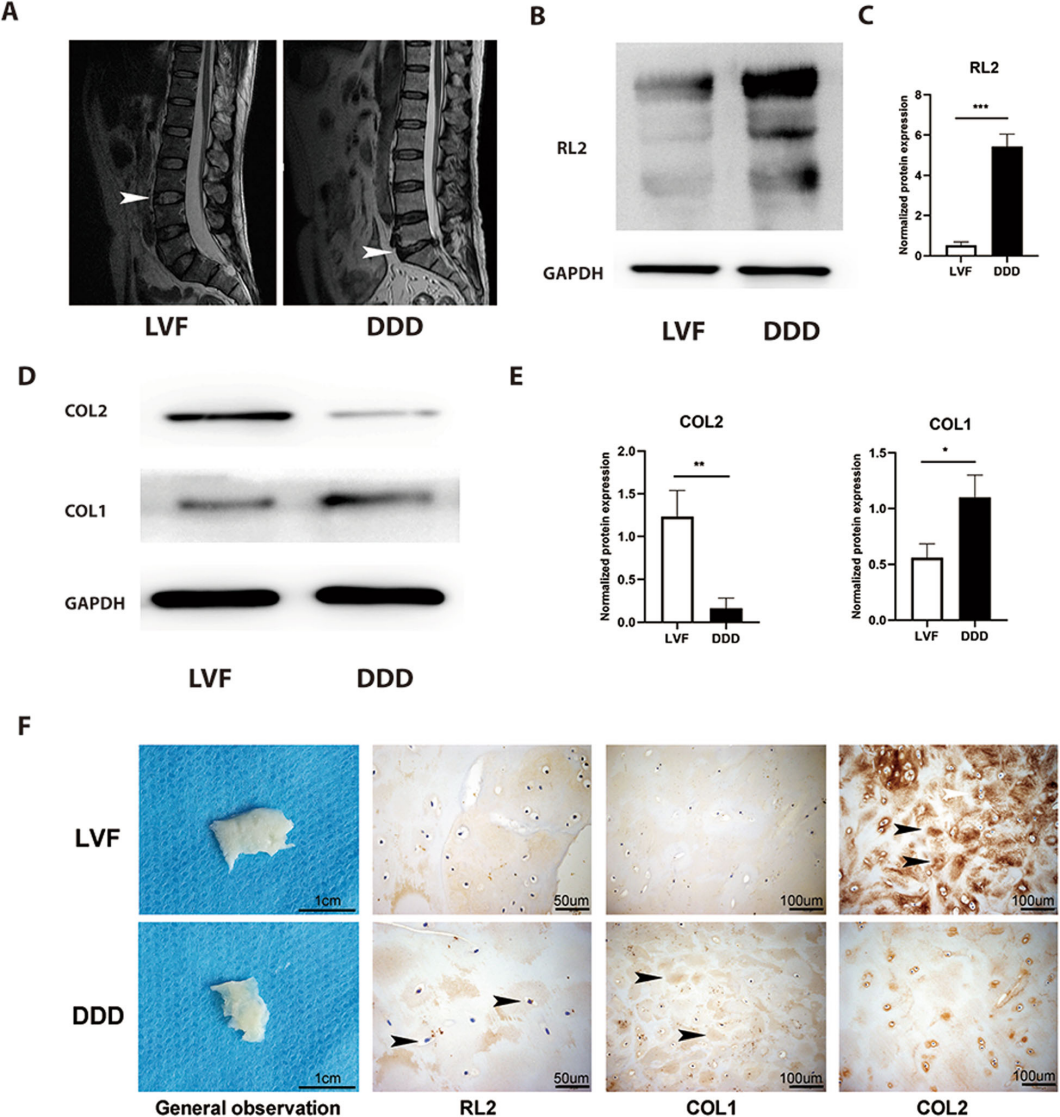
DDD CEPs exposed to normal glucose exhibited less chondrogenesis and more osteogenesis than LVF CEPs. **a** Representative MRI of patients. Patients with LVF were classified as grade 1, and patients with lumbar DDD were classified as grade 6 according to the Pfirrmann classification system. **b**, **c** Western blot (**b**) and analysis (**b**) of protein *O*-GlcNAcylation (RL2). Protein content was measured by densitometry and normalized according to GAPDH level. **d**, **e** Western blot (**d**) and analysis (**e**) of the expressions of COL1 and COL2. The proteins were normalized according to GAPDH level. **f** General observation of samples and immunohistochemical staining of tissue sections. Arrowheads indicate positively stained collagen matrix, cells, or protein. Data represent the mean ± SD (*n* = 3 independent experiments, *t* test). *p < 0.05, **p < 0.01, and ***p < 0.001

To explore the crosstalk in the chondrogenesis, osteogenesis, glucose, and *O*-GlcNAcylation conditions during CEP degeneration, we selected relatively normal CEP tissues from LVF patients as the normal group. At the same time, we selected degenerated CEP tissues from DDD patients as the degenerated group [30] (Fig. 1a). We then used western blot and immunohistochemistry to measure the level of protein *O*-GlcNAcylation and the expression of COL2 and collagen type I (COL1) in CEPs from LVF and DDD patients (Fig. 1b–d). DDD patients’ protein *O*-GlcNAcylation level and COL1 expression were higher than LVF patients. On the contrary, COL2 expression was notably decreased in CEP from DDD patients compared to LVF patients. These results demonstrated that the degenerated CEP of DDD patients lost chondrogenesis while acquired osteogenesis compared to the LVF patients. What is more, the HBP emerged as a glucose sensor [33], and robust *O*-GlcNAcylation is detected in avascular tissues such as the articular cartilage in knee osteoarthritis and nucleus pulposus in degenerated IVD [34]. The increased protein *O*-GlcNAcylation in the CEPs of DDD patients suggested that the degenerated CEP had a higher glucose microenvironment than the normal CEP.

### High glucose inhibited chondrogenesis while promoting osteogenesis and protein *O*-GlcNAcylation

**Fig. 2:**
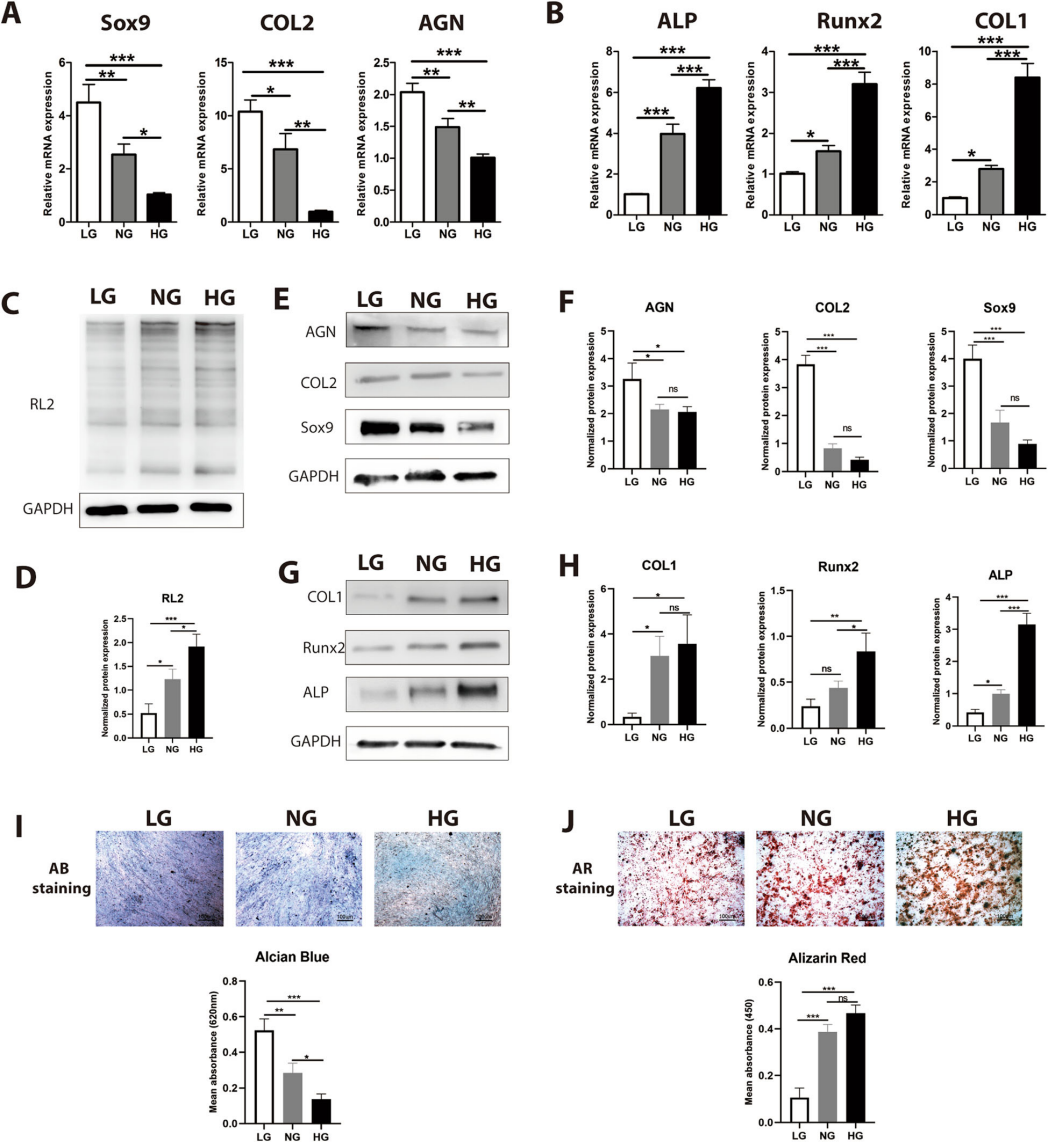
High glucose inhibited chondrogenesis while promoting osteogenesis and protein *O*-GlcNAcylation. CESCs were induced under low-glucose (LG, 1 mM), normal-glucose (NG, 5 mM), and high-glucose (HG, 25 mM) conditions in basic medium (**c**), chondrogenic induction medium (CIM) (**a**, **e**, **i**), or osteogenic induction medium (OIM) (**b**, **g**, **j**), respectively, for 21 days. **a** Expression of chondrogenic genes (Sox9, COL2, and AGN) was assessed by Q-PCR of mRNA from CESCs induced in CIM. **b** Expression of osteogenic genes (Runx2, COL1, and ALP) was assessed by Q-PCR of mRNA from CESCs induced in OIM. **c**, **d** Western blot (**c**) and analysis (**d**) of the protein *O*-GlcNAcylation (RL2) in samples treated under LG, NG, and HG conditions. The protein contents were normalized according to GAPDH level. **e**, **f** Western blot (**e**) and analysis (**f**) of the expressions of Sox9, COL2, and AGN in samples treated under LG, NG, and HG conditions. The protein contents were normalized to GAPDH level. **g**, **h** Western blot (**g**) and analysis (**h**) of the expressions of Runx2, COL1, and ALP in samples treated under LG, NG, and HG conditions. The protein contents were normalized according to GAPDH level. **i**, **j** Macrographs of Alcian blue (AB) staining and Alizarin red (AR) staining and analysis of CESCs treated under LG, NG, and HG conditions. Data represent the mean ± SD (*n* = 3 independent experiments, one-way ANOVA). *p < 0.05, **p < 0.01, and ***p < 0.001

Glucose is the critical energy supply and metabolite for most cells and is especially the key for cell differentiation. To evaluate the influence of glucose on chondrogenic differentiation and osteogenic differentiation, CESCs were induced in chondrogenic induction medium (CIM) and osteogenic induction medium (OIM) under low glucose (LG, 1 mM), normal glucose (NG, 5 mM), and high glucose (HG, 25 mM) [35]. We found that the level of *O*-GlcNAcylation increased from the LG group to the HG group (Fig. 2c). The expression of Sox9, COL2, and AGN decreased from the LG group to the HG group both at the mRNA (Fig. 2a) and protein levels (Fig. 2d). Conversely, the expression of Runx2, ALP, and COL1 increased from the LG group to the HG group both at the mRNA (Fig. 2b) and protein levels (Fig. 2e). Glucose inhibited the formation of chondroitin sulfate and promoted osteogenic mineralization of CESCs (Fig. 2f, g). These results suggested that high glucose is harmful to the normal functions of CESCs. CESCs tend towards osteogenic differentiation rather than chondrogenic differentiation, which could explain why CEP is more calcified when blood vessels grow into it in DDD and disturb the low-glucose environment of CESCs.

### *O*-GlcNAcylation was responsible for high glucose-induced chondro-osteogenic differentiation

**Fig. 3:**
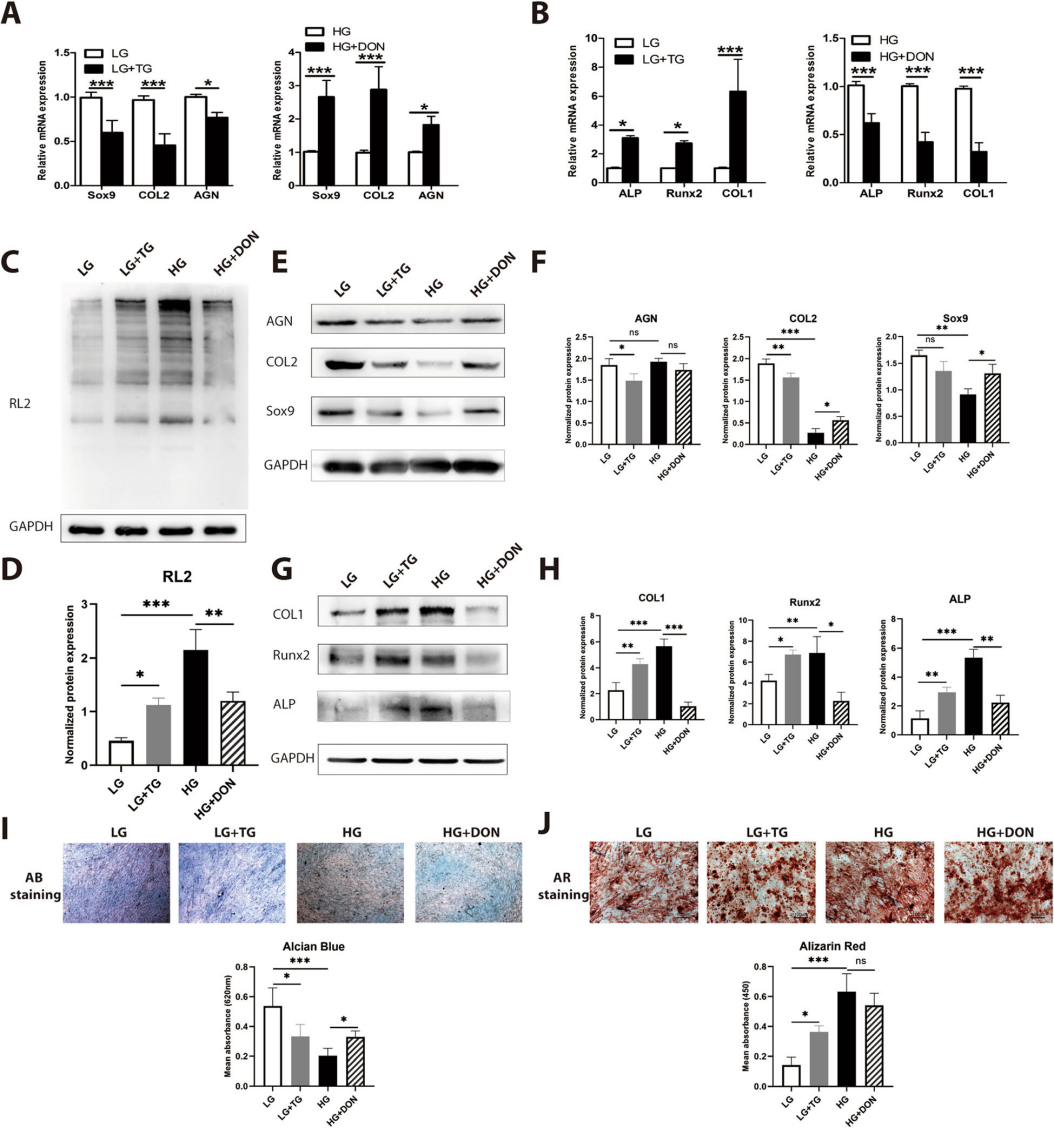
*O*-GlcNAcylation was responsible for high glucose-induced chondro-osteogenic differentiation. CESCs assigned to LG and HG + DON groups, in which the *O*-GlcNAcylation of proteins should be relatively low. CESCs assigned to LG + Thiamet-G (TG) and HG groups in which *O*-GlcNAcylation of proteins should be relatively high. CESCs were induced under low-glucose and high-glucose conditions in basic medium (**c**), chondrogenic induction medium (CIM) (**a**, **e**, **i**), or osteogenic induction medium (OIM) (**b**, **g**, **j**), respectively, for 21 days. **b** Sox9, COL2, and AGN gene expression were assessed by Q-PCR of mRNA from CESCs induced in CIM. **b** Runx2, ALP, and COL1 gene expression were assessed by Q-PCR of mRNA from CESCs induced in OIM. **c**, **d** Western blot (**c**) and analysis (**d**) of protein *O*-GlcNAcylation in each group. The protein contents were normalized according to GAPDH level. **e**, **f** Western blot (**e**) and analysis (**f**) of expressions of Sox9, COL2, and AGN of CESCs induced in CIM. The protein contents were normalized according to GAPDH level. **g**, **h** Western blot (**g**) and analysis (**h**) of expression of Runx2, COL1, and ALP of CESCs induced in OIM. The protein contents were normalized according to GAPDH level. **i**, **j** Macrographs of Alcian blue staining and Alizarin red staining and analysis of CESCs treated under the conditions in **e** and **g**. Data represent the mean ± SD (*n* = 3 independent experiments, one-way ANOVA). *p < 0.05, **p < 0.01, and ***p < 0.001

Glucose conditions in vivo and in vitro affect CESC differentiation, but the underlying mechanisms are poorly understood. *O*-GlcNAcylation controls various physiological processes, such as nutrient sensing, cell cycle progression, stress response, and cell differentiation [24, 36–38]. Therefore, to determine whether *O*-GlcNAcylation contributed to high glucose-induced chondro-osteogenic changes, we used Thiamet-G to increase protein *O*-GlcNAcylation in CESCs and used DON to decrease protein *O*-GlcNAcylation in CESCs. The level of *O*-GlcNAcylation increased in the LT group compared to the LB group, but decreased in the HD group compared to the HB group (Fig. 3c). The expression of chondrogenic differentiation markers (Sox9, COL2, and AGN) decreased and that of osteogenic differentiation markers (Runx2, COL1, and ALP) increased in the LT group compared to the LB group (Fig. 3a, b). The expression of chondrogenic differentiation markers (Sox9, COL2, and AGN) increased and that of osteogenic differentiation markers (Runx2, COL1, and ALP) decreased in the HD group compared to the HB group both at mRNA (Fig. 3a, b) and protein levels (Fig. 3d, e). Glucose inhibited the formation of chondroitin sulfate and promoted osteogenic mineralization of CESCs (Fig. 3f, g). The results above indicated that *O*-GlcNAcylation, a glucose condition converter, regulated CESC chondro-osteogenic differentiation. High levels of protein *O*-GlcNAcylation inhibited chondrogenesis while promoting osteogenesis in CESCs, which is the same effect as the *O*-GlcNAcylation in CEPs of IVD and DDD (Fig. 1b).

### Sox9 and Runx2 were modified by *O*-GlcNAc

**Fig. 4:**
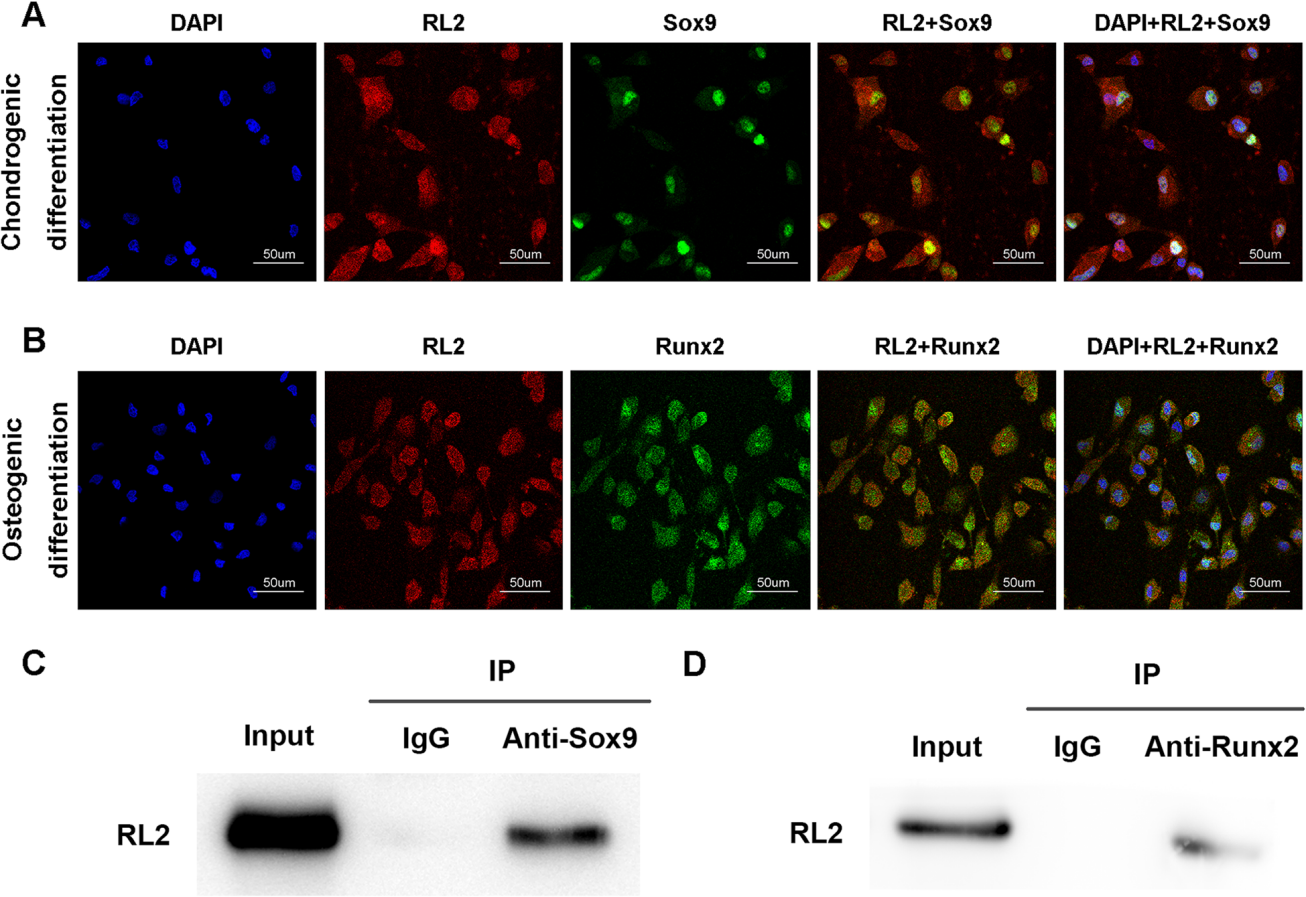
Sox9 and Runx2 were modified by *O*-GlcNAc. CESCs were induced under low-glucose (LG, 1 mM) conditions in chondrogenic induction medium (CIM) (**a**) or osteogenic induction medium (OIM) (**b**), respectively, for 21 days. **a** Immunofluorescence staining of CESCs induced in chondrogenic induction medium for 21 days. **b** Immunofluorescence staining of CESCs induced in osteogenic induction medium for 21 days. **c**, **d** Immunoprecipitation of Sox9 and Runx2 in CESCs induced in CIM and OIM

*O*-GlcNAcylation affects protein function, stability, and localization according to the nutritional status of the cell [39]. To explore the mechanism by which *O*-GlcNAcylation influences CESC fate, we conducted an immunofluorescence analysis after induction. Interestingly, we observed that Sox9 and Runx2 co-localized with *O*-GlcNAcylation in the cell nucleus (Fig. 4a, b). This phenomenon was confirmed by immunoprecipitation; both Sox9 and Runx2 could be modified by *O*-GlcNAc (Fig. 4c, d). Sox9 and Runx2 are core factors in chondrogenic differentiation and osteogenic differentiation processes. This result indicates that *O*-GlcNAcylation may regulate CESC differentiation fate by modifying Sox9 and Runx2.

### *O*-GlcNAcylation promoted Sox9 activity and repressed Runx2 activity

**Fig. 5:**
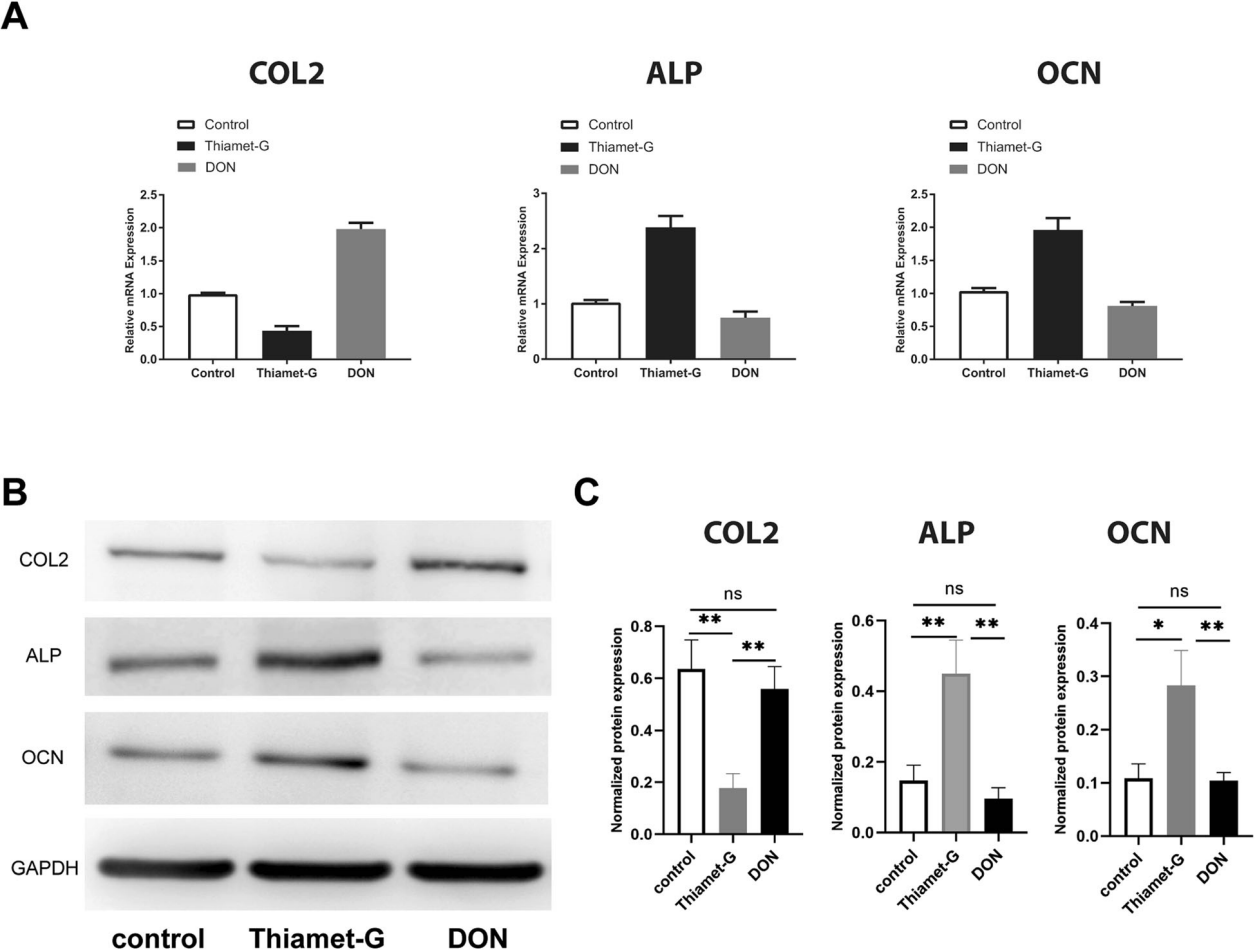
*O*-GlcNAcylation promoted Sox9 activity and repressed Runx2 activity. CESCs assigned to the Thiamet-G group, in which *O*-GlcNAcylation of proteins should be relatively high. CESCs assigned to the DON group in which *O*-GlcNAcylation of proteins should be relatively low. CESCs were induced under low-glucose condition in basic medium for 24 h. **a** COL2, ALP, and OCN gene expressions were assessed by Q-PCR. **b**, **c** COL2, ALP, and OCN protein expressions were assessed and analyzed by western blot. The protein contents were normalized according to GAPDH level. Data represent the mean ± SD (*n* = 3 independent experiments, one-way ANOVA). *p < 0.05, **p < 0.01, and ***p < 0.001

Next, we investigated which *O*-GlcNAcylation modifications of Sox9 and Runx2 influence CESC fate. We used Thiamet-G to increase protein *O*-GlcNAcylation in CESCs and DON to decrease protein *O*-GlcNAcylation in CESCs. We found that the expression of a Sox9 downstream factor (COL2) decreased in the Thiamet-G group but increased in the DON group (Fig. 5a, d). In addition, the expression of Runx2 downstream factors (ALP and OCN) increased in the Thiamet-G group but decreased in the DON group (Fig. 5b–d). These results demonstrate that Sox9 activity is dependent on the *O*-GlcNAcylation level and that *O*-GlcNAcylation of Sox9 inhibits the expression and function of downstream factors, while *O*-GlcNAcylation of Runx2 enhances the expression and function of downstream factors.

## Discussion

In the current study, we demonstrate that glucose in the microenvironment plays an important role in CESC differentiation and that *O*-GlcNAcylation is a key regulator in this process. Glucose is critical for CESC chondro-osteogenic differentiation. Low glucose reduces the potential of CESCs for osteogenic differentiation while increasing chondrogenic differentiation (Fig. 2a–g). Increasing global *O*-GlcNAcylation by Thiamet-G promotes CESC osteogenic differentiation while decreasing its chondrogenic potential, and decreasing O-GlcNAc levels by DON inhibits CESC osteogenic differentiation while increasing its chondrogenic potential (Fig. 3a–f). Together, these results indicate that glucose and *O*-GlcNAcylation are important for both the osteogenic and chondrogenic potential of CESCs. We also demonstrate here that *O*-GlcNAcylation is critical for Sox9 and Runx2 activity and that inhibition of Sox9 and promotion of Runx2 decide the CESC differentiation fate. *O*-GlcNAcylation of Sox9 or Runx2 is the key to influencing many differentiation genes, such as COL2, ALP, and OCN (Fig. 5).

The CEP was free of blood vessels in the group of healthy people without a history of low back pain. However, blood vessel invasion could be observed in people with DDD and IVD [40, 41]. In addition, the physiological low-glucose microenvironment of CESCs was disrupted by blood invasion through small fissures accompanied by increased glucose tension in the microenvironment of degenerated CEP. This disruption of the nutrition supply of CESCs may be a key reason that initiates the loss of chondrogenesis and the acquisition of osteogenesis in CEP. Because of osteogenesis, the CEP loses the capacity to exchange nutrition and resist mechanical stress. Further osteogenesis leads to microenvironment imbalance in the intervertebral disc and initiates IVD degeneration.

CESCs commonly live in a low-glucose environment. Recently, studies have shown that high glucose reduces chondrogenic potential, while the restriction of glucose allows MSCs to maintain chondrogenic differentiation potential [42, 43]. It has also been demonstrated that osteogenic differentiation of MSCs and osteoblasts is stimulated by high glucose [44, 45]. Because *O*-GlcNAc signaling is sensitive to the cell nutritional status, we hypothesized that nutrient status may influence CESC chondrogenic and osteogenic differentiation through *O*-GlcNAcylation. In accordance with this idea, we found that CESCs tended to undergo chondrogenic differentiation in low-glucose conditions while they tended to undergo osteogenic differentiation in high-glucose conditions (Fig. 2).

Cellular energy homeostasis is dependent on the interplay between nutrient-sensing mechanisms and the cellular pathways responsible for energy production. The hexosamine biosynthetic pathway (HBP) is a shunt pathway of glycometabolism that is triggered by increased glucose uptake. HBP is dependent on glucose availability, and decreasing or increasing glucose results in less or more UDP-GlcNAc concentrations, causing different proteins to become more extensively *O*-GlcNAcylated. We speculated that *O*-GlcNAc acts as a nutrient sensor due to the fluctuation of UDP-GlcNAc and protein *O*-GlcNAc levels with the availability of glucose [46].

Previous studies have demonstrated that increased Runx2 *O*-GlcNAcylation contributes to osteoblast differentiation in preosteoblasts and MSCs [47]. The *O*-GlcNAc modification sites on Runx2 are S32, S33, and S371, and *O*-GlcNAcylation of Runx2 could increase transcriptional activity [48]. It has been reported that the O-GlcNAc increases the expression of osteocalcin via regulating Runx2 transcriptional activity in osteogenic differentiation [49]. In this study, we reveal that glucose in the microenvironment regulates the activity of Runx2 via *O*-GlcNAcylation, which is important for the differentiation of CESCs. In high-glucose medium, Runx2 is *O*-GlcNAcylated and target genes (ALP, OCN) of Runx2 are also upregulated (Figs. 4 and 5). Therefore, in DDD, an abnormal glucose macroenvironment induces CESCs towards undergoing osteogenic differentiation, which leads to further aggravate CEP calcification.

It has been reported that the incidence of degenerative disc disease in patients with diabetes and at a younger age is higher than that in the non-diabetic population, which indicates that hyperglycemia contributes to the accelerated degeneration of the intervertebral disc [50]. The *O*-GlcNAcylation of proteins is directly proportional to degeneration [51]. Therefore, *O*-GlcNAcylation caused by an imbalance in the glucose macroenvironment during CEP degeneration may play a key role in CESC differentiation. We first demonstrate that Sox9 was *O*-GlcNAcylated in CESCs (Fig. 4). Sox9 is a major factor in chondrogenic differentiation. COL2, a major cartilage matrix protein, is downstream of Sox9 [52]. We found that COL2 is downregulated in an abnormal glucose environment (Fig. 5). The inhibition of glucose on CESC chondrogenic differentiation promotes CEP calcification and intervertebral disc degeneration occurrence and development.

Our findings uncover the importance of maintaining low-glucose concentration in both in vitro and in vivo microenvironments to support chondrogenesis of CESCs while high-glucose concentration promotes osteogenesis of CESCs, which can potentially benefit the research of disease and tissue engineering, such as DDD induced by hyperglycemic disorders, and offer a viable option to enhance CESC chondrogenesis or osteogenesis by using a pharmacological approach for cartilage or bone regeneration applications. And Sox9 and Runx2 *O*-GlcNAcylation provide a new drug target for DDD.

## Conclusions

**Fig. 6:**
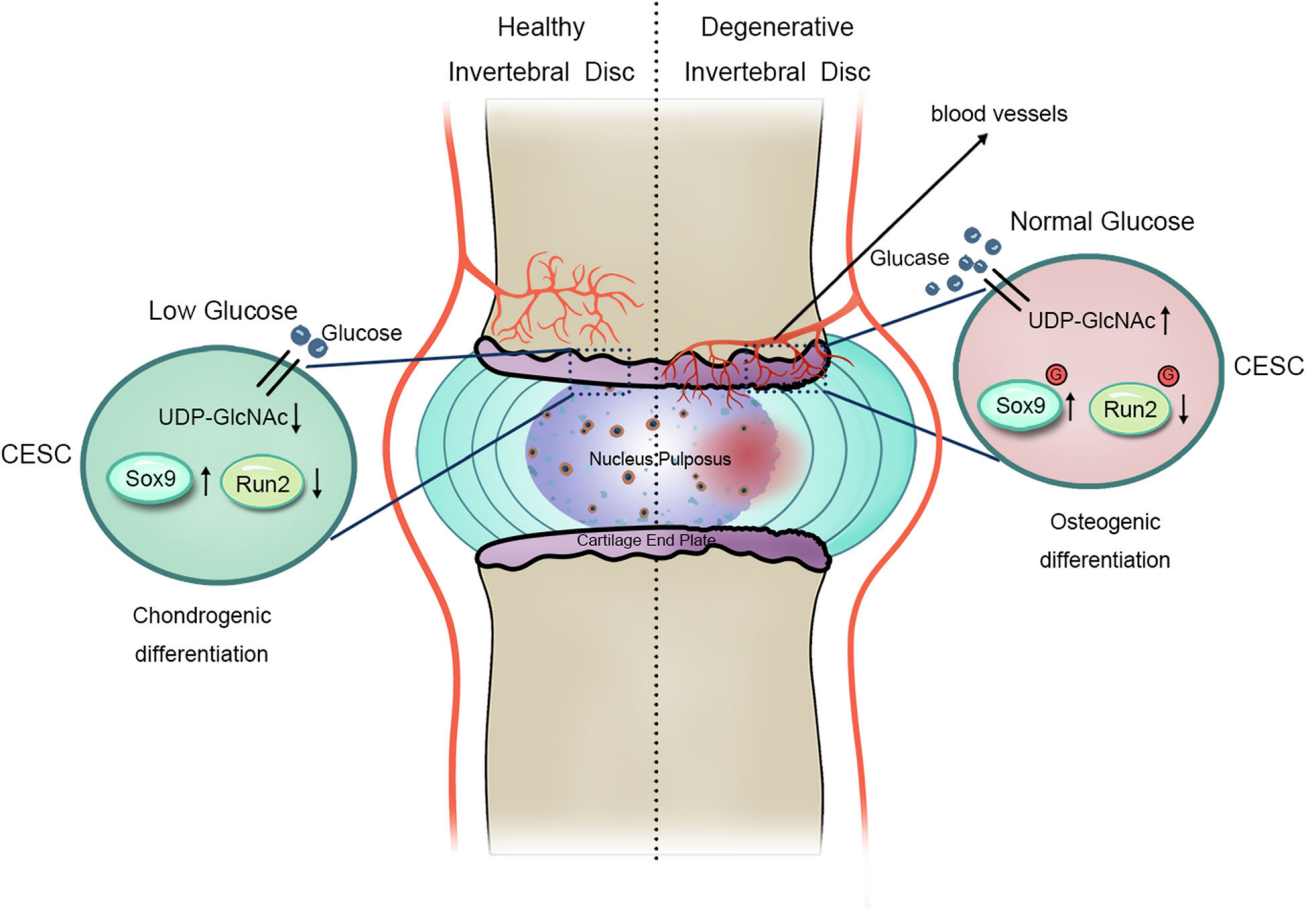
Schematic diagram of proposed mechanisms for glucose-regulated chondro-osteogenic differentiation via *O*-GlcNAcylation in CESCs. In degenerative disc disease, blood vessels grow into cartilage endplate and disturb the glucose environment. Elevated glucose produces more UDP-GlcNAc via the hexosamine biosynthesis pathway. Furthermore, the *O*-GlcNAcylation of Sox9 and Runx2 increases in CESCs with increased UDP-GlcNAc substrate. As a result, increasing *O*-GlcNAcylation modification of Sox9 inhibits Sox9 activity and chondrogenic differentiation, while increasing *O*-GlcNAcylation of Runx2 promotes Runx2 activity and osteogenic differentiation. Ultimately, the CEP shows decreased chondrogenesis and increased osteogenesis, which may aggravate degenerative disc disease

We first identified that glucose regulates human cartilage endplate cell chondro-osteogenic differentiation via *O*-GlcNAcylation. The *O*-GlcNAcylation of Sox9 and Runx2 inhibits CESC chondrogenic differentiation and promotes osteogenic differentiation. Our findings suggest that the glucose-induced *O*-GlcNAcylation of Sox9 and Runx2 in CESCs may be a target for CEP degeneration therapy (Fig. 6).

## Supplementary information

**Additional file 1: Figure S1.** CESCs Shared Features with BM-MSCs Regarding Morphology, Stem Cell Surface Markers, and Differentiation Ability.

## Data Availability

All data generated or analyzed during this study are included in this published article.

## Abbreviations

BM-MSCs: Bone marrow mesenchymal stem cells
CEP: Cartilage endplate
CESCs: Cartilage endplate stem cells
CIM: Chondrogenic induction medium
COL1: Collagen type I
COL2: Collagen type II
DDD: Degenerative disc disease
GlcNAc: *N*-acetylglucosamine
HBP: Hexosamine biosynthetic pathway
HG: High glucose
IVD: Intervertebral disc
LG: Low glucose
LVF: Lumbar vertebral fracture
NG: Normal glucose
OGA: *O*-GlcNAcase
O-GlcNAc: *O*-linked β-*N*-acetylglucosamine
OGT: *O*-GlcNAc transferase
OIM: Osteogenic induction medium
Runx2: Runt-related transcription factor 2
