# Supplementary material for: Glucose regulates tissue-specific chondro-osteogenic differentiation of human cartilage endplate stem cells via O-GlcNAcylation of Sox9 and Runx2
Source: Stem Cell Res Ther. 2019 Nov 28;10:357. doi: 10.1186/s13287-019-1440-5 (PMC6883626; doi:10.1186/s13287-019-1440-5)
Supplement: Supplementary file 1 — Additional file 1: Figure S1. CESCs Shared Features with BM-MSCs Regarding Morphology, Stem Cell Surface Markers, and Differentiation Ability. [file 13287_2019_1440_MOESM1_ESM.docx]

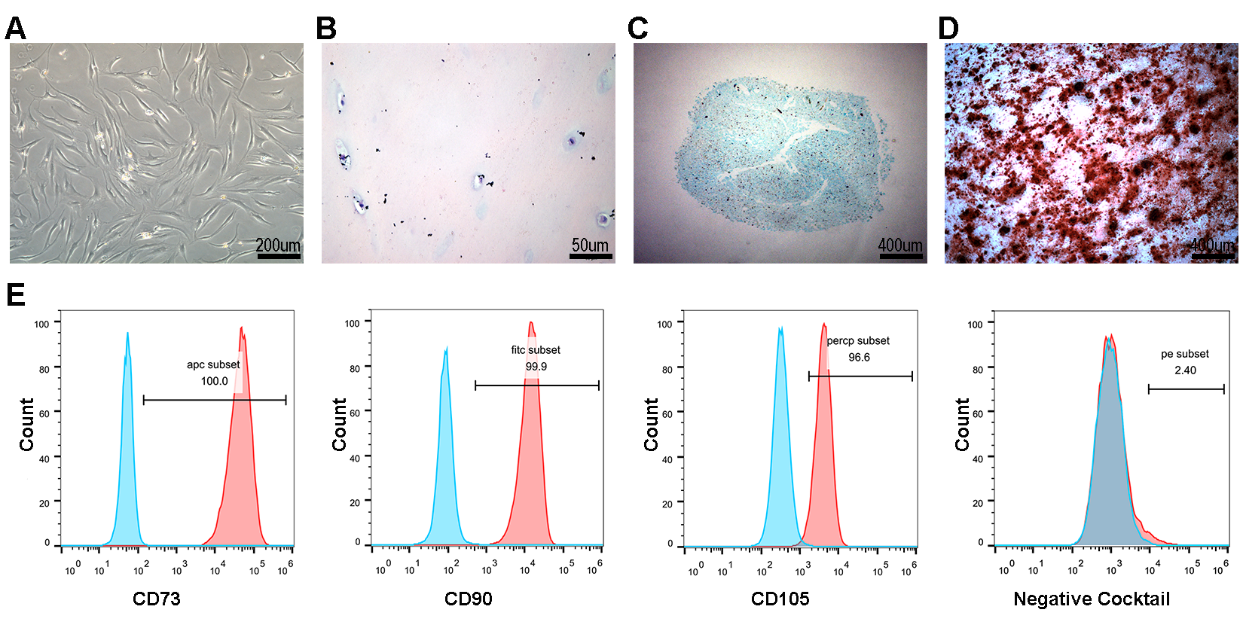


**Additional file 1: Figure S1.** CESCs Shared Features with BM-MSCs Regarding Morphology, Stem Cell Surface Markers, and Differentiation Ability.

**A:** Morphology of CESCs 4 weeks after seeding. **B:** H&E staining of tissue sections. **C:** Histological section stained with Alcian blue of chondrified masses formed by CESCs after 3 weeks of incubation with chondrogenic induction medium. **D:** Alizarin red staining of CESCs that underwent osteogenic induction for 3 weeks. **E:** Immunophenotypic stem cell profile of CESCs by flow cytometric analysis.
